# Associations between patterns of physical activity, pain intensity, and interference among older adults with chronic pain: a secondary analysis of two randomized controlled trials

**DOI:** 10.3389/fragi.2023.1216942

**Published:** 2023-07-25

**Authors:** Jason Fanning, Amber K. Brooks, Justin T. Robison, Megan B. Irby, Sherri Ford, Kindia N’Dah, W. Jack Rejeski

**Affiliations:** ^1^ Department of Health and Exercise Science, Wake Forest University, Winston-Salem, NC, United States; ^2^ Department of Anesthesiology, Wake Forest University School of Medicine, Winston-Salem, NC, United States

**Keywords:** pain, physical activity, behavior, aging, accelerometry

## Abstract

**Background:** Clinical management of chronic pain often includes recommendations to engage in physical activity (PA), though there are little data on the interplay between pain symptoms and key aspects of PA participation (e.g., intensity and bout duration) among older adults. Herein we investigate the longitudinal relationships between changes in PA behavior and changes in pain intensity and interference among low-active older adults with obesity and chronic pain.

**Methods:** Participants (*N* = 41) were enrolled in two randomized pilot trials wherein they were assigned to an intervention focused on participation in frequent PA across the day, or to a low-contact control. Participants completed the 3-item PROMIS pain intensity scale and 8-item PROMIS pain interference scale before and after the interventions. Participants also wore an ActivPAL accelerometer for 7 days before and during the final week of the interventions.

**Results:** A series of linear regression analyses demonstrated that increased time spent stepping at a high-light intensity in very short bouts was associated with increased pain intensity scores. By contrast, increased time spent stepping at a high-light intensity in bouts of 5–20 min was associated with reductions in pain intensity and interference scores. Increased time spent stepping at a moderate intensity overall was associated with reduced pain intensity scores, and time spent stepping at a moderate intensity for 10–20 min associated with reduced pain interference.

**Conclusion:** These findings suggest older adults with chronic pain may benefit by moving at high-light or moderate intensities in brief bouts of at least 5 min in duration.

## 1 Introduction

The experience of chronic pain is common in older adulthood, is socioeconomically burdensome, and affects quality of life and wellbeing (Domenichiello and Ramsden, 2019). Clinical management of chronic pain often includes recommendations to engage in physical activity (PA), (Antcliff et al., 2022), as overall levels of PA are often associated with reduced risk for chronic pain (Nijs et al., 2020). Pain and PA have reciprocal effects, with pain acting as a potent barrier to regular participation in PA, (Stubbs et al., 2013; Stubbs et al., 2014), potentially increasing pain symptoms alongside myriad other negative physical and psychological health consequences of insufficient physical activity (Physical Activity Guidelines Advisory Committee, 2018; Piercy et al., 2018). Evidence from observational and experimental trials suggests that regularly participating in light and moderate-intensity PA is associated with improvement in pain symptoms, while higher levels of intensity can benefit pain symptoms among those who will tolerate it (Ambrose and Golightly, 2015). Furthermore, the relationship between PA and pain is nuanced and subjective, as the impact of PA on pain symptoms varies based on PA intensity, modality, bout length (e.g., few long bouts vs. frequent short bouts), and perceived enjoyment. To date, there are limited objective data on the interplay between pain symptoms (intensity and interference) and PA intensity and bout duration among older adults. Herein we present an analysis of PA behavior assessed objectively using ActivPAL accelerometers among older adults with chronic pain. Participants were involved in two distinct randomized controlled pilot studies: the 12-week *Mobile Intervention to Reduce Pain and improve Health* (MORPH) and MORPH-II, (Fanning et al., 2020; Fanning et al., 2022a), which was a similar intervention with refinements based on knowledge gleaned in MORPH.

As noted above, the relationship between PA and pain is complex. Laboratory data suggest that greater time spent in moderate-to-vigorous intensity PA (MVPA) is a strong predictor of pain facilitation (i.e., greater MVPA is associated with less temporal summation of pain), while less sedentary time and more light intensity physical activity (LPA) are associated with greater pain inhibitory capacity (Naugle et al., 2017). It is notable that sedentary time and LPA have a strong inverse relationship with one another, are driven by habit, and occur in higher volumes across the day relative to MVPA (Owen et al., 2010; Dunstan et al., 2012; Fanning et al., 2022a). Thus, it is logical that these PA behaviors yield different effects on various measures of pain. For example, it is widely suspected that there is a U-shaped relationship between pain and PA intensity such that very low or very high intensity PA worsens pain symptoms (Heneweer et al., 2009; Ambrose and Golightly, 2015). One common clinical PA recommendation for those with chronic pain centers on *activity cycling*, though this term is often used to describe very different behaviors. One common use of the term describes a strategy for enhancing participation in PA by engaging in brief bouts of activity more often during the day, thus avoiding overexertion and hyperalgesia. (Nielson et al., 2013). This recommendation was supported by a recent meta-analysis (Polaski et al., 2019) examining physical activity dosing parameters (i.e., frequency, time, and duration) and change in pain among individuals with chronic pain. The authors found that increasing frequency of activity was associated with improved pain symptoms. Likewise, this recommendation also aligns well with recent current PA guidelines that emphasize moving more and more often (Physical Activity Guidelines Advisory Committee, 2018; Piercy et al., 2018).

Currently, limited objective data exist on the interplay between the pattern of PA accumulation (i.e., bout length), intensity of movement, and pain symptoms in older adults. The MORPH and MORPH-II randomized pilot trials offer an excellent opportunity to explore these relationships. In each of these trials, older adults wore ActivPAL PA monitors, which provide excellent classification of sedentary (Rosenberg et al., 2020) and stepping (Wu et al., 2022) behaviors. Participants also completed measures of pain intensity and pain interference before and after participating either a 12-week group-mediated dietary weight loss and PA program focused on moving throughout the day or a 12-week control group. The purpose of this *post hoc* exploratory analysis is to investigate the longitudinal relationships between changes in PA behavior (i.e., sedentary time, light and moderate-intensity stepping accrued in bouts of varying durations) and changes in pain intensity and pain interference among low-active older adults with chronic pain. We hypothesized that increasing time spent in activities near moderate intensity PA (i.e., high levels of light PA and moderate-intensity walking), especially in more frequent brief bouts, would relate to improvements in participant-reported pain intensity and pain interference. Conversely, greater sedentary time, fewer sedentary breaks, and sustained participation in more intense activity would be related to higher pain intensity and pain interference scores.

## 2 Materials and methods

The institutional review boards at Wake Forest University School of Medicine and Wake Forest University approved all study-related procedures for MORPH and MORPH-II respectively. Both trails were registered at ClinicalTrials.gov (MORPH: NCT03377634; MORPH-II: NCT04655001).

### 2.1 Participants

Individuals included in this analysis participated in the MORPH(9) or MORPH-II(19) randomized controlled pilot trials that investigated the impact of a group-mediated behavioral intervention focused on movement across the day and caloric restriction on pain among older adults with chronic pain (described further below). The procedures and primary outcomes of each study have been reported previously (Fanning et al., 2018; Fanning et al., 2020; Fanning et al., 2021a; Fanning et al., 2022b). Briefly, recruitment for MORPH occurred between 2018 and 2019, and MORPH-II recruitment occurred between 2021 and 2022. For both studies, eligible individuals were aged 55–85, had a body mass index (BMI) of 30–45 kg/m^2^ [self-reported in MORPH-II and corrected via the Shields equation (22)], self-reported to be low-active, self-reported to be weight stable, and had no contraindication to exercise. Additional requirements for MORPH included the ability to attend the research center for testing and group sessions, and access to a smartphone and willingness to engage with a study-specific smartphone app. MORPH participants were also required to have self-reported chronic pain in at least 2 of the following sites on most days in the previous 12 weeks: neck, shoulder, back, hip, or knee. For MORPH-II, participants were provided a tablet computer for the duration of the study to access remote group videoconference meetings. Participants in MORPH-II were not required to attend any in-person sessions, and the pain inclusion criterion changed to self-reported chronic pain in at least one of the following sites on most days in the previous 12 weeks: back, hip, or knee.

### 2.2 Interventions

#### 2.2.1 Measurement control

Participants randomized to the measurement control condition received a Fitbit Inspire accelerometer and a BodyTrace cellular-enabled scale (BodyTrace, Inc., 2022). This enhanced usual care control was designed to account for any potential effect of device provision on health behaviors such as physical activity or diet.

#### 2.2.2 The MORPH group-mediated behavioral intervention

MORPH and MORPH-II were iterative refinement trials centered on designing a novel intervention originally focused on increasing PA via movement throughout the day paired with caloric restriction to reduce pain symptoms (Fanning et al., 2018; Fanning et al., 2020). In each study participants were recruited in waves (3 in MORPH and 5 in MORPH-II) so that minor refinements could be inventoried, implemented, and tested between waves. Lessons learned from MORPH were published (Fanning et al., 2020) and incorporated into a new protocol for MORPH-II (Fanning et al., 2021a). Key changes included: 1) a reduced focus on caloric restriction and greater on engaging in PA across the day; 2) the implementation of a technology kit allowing all eligible individuals to participate regardless of device ownership or access to high-speed internet; 3) a shift toward fully-remote delivery as participants disliked the process of changing from in-person to videoconference sessions (MORPH began with three in-person sessions to attempt to form strong group bonds); and 4) incorporation of brief coaching contacts with trained student coaches to enhance understanding of personal activity patterns. These calls followed a tapered schedule such that up to three weekly calls were made at the start of the program, and this frequency was reduced to up to one weekly call by week six.

The MORPH interventions have been described in detail elsewhere (Fanning et al., 2018; Fanning et al., 2020; Fanning et al., 2021a; Fanning et al., 2022b). Briefly, participants engaged in a group-mediated program informed by social cognitive theory, (Bandura, 1997) self-determination theory, (Deci and Ryan, 2008) mindfulness-based relapse prevention, (Bowen et al., 2009) and principles of group dynamics (Brawley et al., 2014). Participants met in groups of approximately 4-8 individuals once per week under the guidance of a trained behavioral interventionist. The group structure itself acted as a key tool of behavior change, allowing for development of social bonds, modeling of successes, troubleshooting challenges as they arose, and collaboration on programmatic goals. Given that pain arises through the interaction of physical and cognitive/emotional inputs, a major focus of the MORPH interventions was mindfulness utilizing strategies from mindfulness-based relapse prevention, (Bowen et al., 2009), a framework that unites principles from the relapse prevention model (Marlatt, 1989) (a derivative of social cognitive theory) and mindfulness.

Outside of weekly hour-long group sessions, participants engaged with a *Companion App* mHealth toolset that was developed by one member of the research team (JF) across four randomized trials (Fanning et al., 2017; Fanning et al., 2020; Fanning et al., 2022a; Fanning et al., 2022b). This toolset, which integrated data in real time from participants’ Fitbit Inspire activity monitors and BodyTrace scales, fulfilled three important behavioral support roles: 1) it allowed for ongoing communication via asynchronous chat between group members between meetings; 2) it provided objective visual and numeric feedback on the amount and patterning of PA via real-time Fitbit data streamed into the app, facilitating better awareness of activity patterns and providing a platform for pattern-based goal setting; and 3) it supported the development of self-efficacy by highlighting goal successes via badges; participants were trained to use these badges as cues to savor their progression.

There were four key changes between MORPH and MORPH-II based upon lessons learned in MORPH. First, MORPH-II reduced its focus on caloric restriction and more strongly emphasized moving across the day. Second, where MORPH included both in-person and remote sessions, MORPH-II was delivered fully remotely. This allowed participation among individuals who were geographically or socially isolated and facilitated conduct of the intervention during the COVID-19 pandemic. Third, MORPH-II provided technology kits including an iPad (cellular enabled for those without home internet), the Fitbit, and wireless weight scale, with all devices prepared for use out-of-the-box. This allowed for participation by those without access to smartphone or tablet technology. Finally, to further support early uptake of the daylong activity recommendation, trained student behavioral coaches completed brief coaching calls in the early weeks of MORPH-II with a focus on reviewing daily activity pattern feedback and revising weekly goals. We previously reported that the MORPH intervention contributed to several beneficial effects, including reduced pain intensity and sedentary time, though there was little improvement in daily steps (Fanning et al., 2020; Fanning et al., 2021b). MORPH-II also yielded a decrease in pain intensity, though the control group also demonstrated a sizable decrease in pain intensity. As such, the effect size between conditions favored the intervention condition but was small in magnitude. MORPH-II more powerfully affected PA behavior, contributing to large magnitude effects on steps and sedentary breaks relative to control. The MORPH-II intervention also yielded beneficial moderate-sized effects on measures of autonomy, relatedness, and competence relative to control (Fanning et al., 2022b).

### 2.3 Measures

#### 2.3.1 Demographics

Participants self-reported demographics including date of birth, sex, and race prior to the start of the program.

#### 2.3.2 Physical activity

Participants in both MORPH and MORPH-II wore an ActivPAL 4 triaxial accelerometer (PAL Technologies, Glasgow, Scotland). This small accelerometer is worn on the midline of the thigh and provides excellent classification of stepping and sedentary behaviors (Rosenberg et al., 2020; Wu et al., 2022). The monitor was worn for 7 consecutive days on the non-dominant leg prior to the start of the intervention and during the final week of the intervention. Data from MORPH and MORPH-II were processed using PALBatch version 8.11.63 following a 24-h wear protocol (i.e., a minimum of 20 h of wear time was required for a valid day) and data were classified using the CREA algorithm (version 1.3), with minimum threshold for distinguishing up-right and non-upright periods set to 10 s (Edwardson et al., 2017). Individuals with fewer than 3 valid days of wear at either time point were excluded from analyses. Outcomes of interest included average daily steps, average daily minutes spent sedentary (i.e., low-active in a seated or lying posture), and number of sedentary breaks (i.e., postural shifts from sitting to standing). There are no validated acceleration threshold cut points for the ActivPAL activity monitor. Instead, the device utilizes stepping cadence bands to estimate time spent ambulating at different intensities. It is notable that one advantage of the ActivPAL is that it distinguishes between stepping and non-stepping behaviors (Wu et al., 2022), whereas accelerometers that score using acceleration thresholds (e.g., ActiGraph devices) do not, though their scoring thresholds are calibrated to stepping behavior. As such, many non-ambulatory behaviors such as home chores may be inappropriately categorized. (Fanning et al., 2022c). The present analyses are exploratory in nature, with the purpose of better understanding the longitudinal effects of movement intensity and bout duration on pain intensity and interference. Therefore, we leveraged a series of stepping thresholds proposed by Tudor-Locke and colleagues (Tudor-Locke et al., 2021). These researchers demonstrated that a threshold of 100 steps per minute differentiates light vs. moderate intensity activity in older adults. A threshold of 125 steps per minute approximates the highest threshold Tudor-Locke and colleagues observed (128 steps/minute; 5 METs), which corresponds to high-moderate activity. Additionally, we utilized a threshold of 75 steps/minute to distinguish between very-light and light intensity ambulation. We investigated average daily steps and time spent stepping at a rate of <75 steps/minute (hereafter “very-light”), 75–100 steps/minute (hereafter “light”), and 100–125 steps/minute (hereafter “moderate”). We also investigated time spent above 125 steps per minute, but participants very rarely accrued time in this range and it was therefore dropped from the analyses. Finally, to explore the effect of bout duration within intensity categories, we further investigated both average daily steps and time spent in each cadence band in the following bout durations: <1 min, 1–5 min, 5–10 min, 10–20 min, and greater than 20 min.

#### 2.3.3 Pain intensity and interference

Pain intensity, or the magnitude of one’s experience of pain, was assessed using the 3-item PROMIS pain intensity scale (version 2). Pain interference, which captures the extent to which pain interferes with daily life, was measured using the 8-item PROMIS pain interference scale. (HealthMeasures, 2017). Both scales were scored using the PROMIS scoring system, which produces standardized scores wherein 50 represents the national average, with 10 points as the standard deviation. For each scale, higher scores indicate greater pain intensity or pain interference.

### 2.4 Analyses

Descriptive characteristics, including mean ± standard deviation (SD) for continuous variables and frequencies and percentages for count variables, were computed for participant characteristics. A series of linear regression models were utilized to assess relationships between changes in PA behavior and changes in pain intensity or pain interference. In each model, pain intensity or pain interference at week 12 was included as the dependent variable, with scores at baseline included as a covariate. Additional covariates in each model included participant age, group assignment to account for any impact of the MORPH intervention on pain outside of activity behavior, sex, and study to account for any differential effects of MORPH and MORPH-II. For models investigating stepping time, we included total stepping time (the ActivPAL proxy for total activity time) at baseline and change in stepping time to investigate relationships between how one distributes their movement (i.e., intensity and bout length) while controlling for total amount of movement. A new model was run for each physical activity variable, including change in the variable over 12 weeks and the baseline score. Finally, because of the exploratory nature of these analyses, we did not adjust for multiple comparisons as we deem a type-II error to be at least as impactful as a type-I error, (Rothman, 1990), and as such we instead emphasize the exploratory nature of the analyses. All models were checked to ensure there was no multicollinearity and that residuals were relatively normally distributed. SPSS version 28 (IBM incorporated, Armonk, NY) was used for all analyses.

## 3 Results

In total, 75 individuals wore an ActivPAL accelerometer in the context of the MORPH and MORPH-II studies. Of these, 41 participants across both studies had sufficient ActivPAL data for inclusion in analyses, with the primary reason for data loss being battery failure (see limitations for additional detail). Demographic and baseline characteristics did not differ between those with and without sufficient data for inclusion (Supplemental Table S1). Participant demographics and baseline activity and pain data are displayed in Table 1 with additional descriptive information related to activity bout duration provided in Supplementary Table S2. The average age of the sample was 69.61 ± 6.48 years, and participants predominantly identified as female (73%), white (81%), and college educated (88%). Participants had 6.32 ± 1.19 and 6.17 ± 1.76 days of valid accelerometer data on average at baseline and week 12 respectively. On average, participants reported pain intensity and pain interference that were each approximately one standard deviation above national averages (59.44 ± 6.59 and 59.82 ± 6.24 respectively). Participants achieved 4828.58 ± 2627.89 steps per day on average, which is well below current older adult-specific step translations of the US physical activity guidelines. (Tudor-Locke et al., 2011). Participants engaged in sedentary behaviors for more than 11 h daily (686.66 ± 134.58 min/day) and in stepping behaviors for just over 1 hour daily (67.05 ± 35.70 min/day). The majority of time spent in PA was accrued at very-light intensity (35.97 ± 21.50 min/day) and light intensity bouts (25.37 ± 14.38 min/day), with only 6.38 ± 7.10 min/day spent in moderate intensity.

**TABLE 1:** Participant Information at baseline. Notes: M, mean; SD, standard deviation; physical activity data represent averages across valid days of wear. Very light intensity time = average daily minutes spent stepping at <75 steps/minute (s/m); light intensity time = average daily minutes spent stepping at 75–100 s/m; moderate intensity time = average daily minutes spent stepping at 100–125 s/m.

	MORPH (*n* = 20)	MORPH-II (*n* = 21)	Overall (*N* = 41)
Age, yrs; M(SD)	69.84 (5.18)	69.38 (7.65)	69.61 (6.48)
Pain Intensity; M(SD)	59.48 (8.38)	59.42 (4.48)	59.44 (6.59)
Pain Interference; M(SD)	63.44 (4.23)	56.37 (5.94)	59.82 (6.24)
Intervention; n(%)	11 (55.00)	12 (57.10)	23 (56.10)
Male; n(%)	5 (25.00)	6 (28.60)	11 (26.80)
White; n(%)	17 (85.00)	16 (76.20)	33 (80.50)
College Educated; n(%)	16 (80.00)	20 (95.24)	36 (87.80)
Weight, kg; M(SD)^a^	101.69 (16.45)^b^	95.55 (12.01)	98.70 (14.61)
Steps; M(SD)	4594.37 (2688.21)	5051.64 (2615.23)	4828.58 (2627.89)
Stepping Time; M(SD)	63.09 (35.45)	70.82 (36.39)	67.05 (35.70)
Sedentary Time; M(SD)	722.53 (147.77)	652.5 (113.87)	686.66 (134.58)
Breaks; M(SD)	41.16 (11.51)	47.44 (15.01)	44.38 (13.62)
Very Light Intensity Time; M(SD)	32.18 (17.11)	39.58 (25.05)	35.97 (21.60)
Light Intensity Time; M(SD)	25.32 (15.04)	25.41 (14.1)	25.37 (14.38)
Moderate Intensity Time; M(SD)	6.92 (7.76)	5.86 (6.57)	6.38 (7.10)

^a^ Data available on *n* = 39.

^b^ Data available on *n* = 19.

Table 2 describes results for the series of linear regressions relating changes in activity variables to change in pain intensity or pain interference over 12 weeks. Notably, in this sample of low-active older adults with chronic pain, there were insufficient data on time spent ambulating at a very-light intensity for more than 20 min or at intensities greater than 125 steps per minute to fit regression models.

**TABLE 2:** Relationship between change in physical activity behaviors and pain intensity or interference. Each model includes as covariates: baseline pain intensity or interference, baseline for the relevant change score listed here, age, sex, study, group assignment, total activity time at baseline, and change in total activity time. Very light intensity time = average daily minutes spent stepping at <75 s/m; light intensity time = average daily minutes spent stepping at 75–100 s/m; moderate intensity time = average daily minutes spent stepping at 100–125 s/m.

	Pain intensity			Pain interference		
	*B*	*t*	*p*	*B*	*t*	*p*
Steps	<0.001	−.067	.947	.001	.614	.544
Stepping Time	−.006	−.091	.928	.074	.957	.346
Sedentary Time	.003	.250	.804	−.012	−.858	.397
Sedentary Breaks	−.061	−.399	.693	−.027	−.156	.877
Very Light Intensity Time; M(SD)	−.091	−.551	.585	.185	.991	.329
<1 min	−.078	−.402	.690	.238	1.080	.289
1–5 min	−.276	−.426	.673	.259	.369	.714
5–10 min	−3.146	−1.299	.203	−.305	−.105	.917
10–20 min	−1.640	−.379	.707	1.330	.261	.796
20+ minutes^a^	—	—	—	—	—	—
Light Intensity Time; M(SD)	**.513**	**2.492**	.018*	−.369	−1.419	.166
<1 min	**.954**	**3.130**	.004*	.501	1.272	.213
1–5 min	**.663**	**2.205**	.035*	−.558	−1.533	.135
5–10 min	−.387	−.652	.519	**−1.299**	**−2.231**	.033*
10–20 min	**−1.915**	**−2.165**	.038*	−1.419	−1.329	.193
20+ minutes	−1.089	−1.373	.179	−1.359	−1.504	.143
Moderate Intensity Time; M(SD)	**−.429**	**−2.277**	.030*	.025	.106	.916
<1 min	.312	.438	.664	1.503	1.825	.078
1–5 min	−.045	−.066	.948	−.725	−.892	.379
5–10 min	−.969	−.801	.429	1.721	1.339	.190
10–20 min	−1.374	−1.645	.110	**−2.129**	**−2.345**	.026*
20+ minutes	−1.143	−1.618	.116	−.712	−.813	.422

^a^ Insufficient observations.

Statistically significant (*p* < .05) predictors are noted in bold.

Baseline-adjusted change in average daily steps, stepping time, sedentary time, and breaks in sedentary time were not related to baseline-adjusted change in pain intensity (*p*s ≥ .693) or pain interference (*p*s ≥ .346). Likewise, adjusted change in overall time spent stepping either at a very-light intensity or in any bout duration was not associated with adjusted change in pain intensity (*p*s ≥ .203) or interference (*p*s ≥ .289). Increasing overall time spent at a light intensity, or in bouts of up to 5 min was associated with increases in pain intensity (*p*s ≤ .035). By contrast, engaging in bouts of 10–20 min was associated with less pain intensity. Specifically, for each additional minute spent in a bout of 10–20 min, pain intensity decreased by 1.92 points (*p* = .038). Regarding pain interference, engaging in brief bouts (5–10 min) of light intensity activity was associated with less interference such that for each addition minute of PA, pain interference scores decreased by 1.30 points (*p* = .033).

Overall, moderate intensity stepping was associated with less pain intensity such that each additional minute of moderate stepping was associated with a 0.43-point decrease in pain intensity (*p* = .030). Furthermore, time spent moving at a moderate intensity for 10–20 min was associated with improvement in pain interference (*p* = .026). Specifically, each additional minute spent moving at a moderate intensity in bouts of 10–20 min was associated with a 2.13-point decrease in pain interference.

## 4 Discussion

This study explored the longitudinal relationships between changes in PA behavior with pain intensity and pain interference among low-active older adults with chronic pain enrolled in the MORPH and MORPH-II pilot studies. We hypothesized that increasing time spent in activities near moderate intensity PA (i.e., high levels of light PA and moderate-intensity walking), especially in more frequent brief bouts, would relate to improvements (i.e., reductions) in pain intensity and pain interference scores. Conversely, we hypothesized that greater sedentary time, fewer sedentary breaks, and sustained participation in more intense activity would be related to increased pain intensity and pain interference scores. Finally, we hypothesized that time spent engaging in intense activity would be associated with worse symptoms, though we were unable to evaluate this hypothesis, as very few participants engaged in this level of activity.

Results partially supported our hypotheses. We observed that after controlling for group assignment, study, age, and sex, stepping at a light intensity for 10 min, or stepping at a moderate intensity in general was associated with decreases in pain intensity over 12 weeks. Engaging in 5–20-min bouts of light and moderate-intensity walking was associated with reduced pain interference. Counter to our hypotheses, change in daily steps, stepping time, sedentary time, and sedentary breaks were not associated with changes in pain intensity or interference. Interestingly, when individuals spend more of their overall movement time in short, light-intensity bouts they were more likely to report increases in pain intensity between baseline and 12 weeks.

It is intriguing that individuals who spent more of their time moving in very short (i.e., <5 min) bouts at a light intensity demonstrated increasing pain intensity, given that an explicit goal of MORPH and MORPH-II was to achieve greater volumes of movement across the day, irrespective of bout duration. Evidence to date does not suggest that short bouts are less effective than long bouts of PA in managing pain intensity (Parsons et al., 2017; Jakicic et al., 2019; Giurgiu et al., 2020), though it is notable that short activity bouts are typically defined as <10 min in duration (note that moving for 5–10 min was not associated with pain intensity). Conversely, our findings align well with evidence on activity fragmentation, a phenomenon whereby spending more time in very short bouts of movement (often defined as those lasting <5 min) may reflect compensatory changes in response to impaired health states (Wanigatunga et al., 2019a). For instance, an individual with impaired functional capacity may require breaks more often while completing core activities of daily living. Greater activity fragmentation is associated with increased risk of all-cause mortality among older adults, (Wanigatunga et al., 2019a), and cancer survivors tend to engage in more fragmented activity relative to adults without a history of cancer (Wanigatunga et al., 2018). Thus, greater activity fragmentation may represent a useful clinical tool that characterizes a phenotype of older pain patients who are more strongly affected by their pain symptoms, perhaps due to the nature of the disease state(s) underlying their pain.

Aside from the potential clinical value of the activity fragmentation metric, our results also provide a framework for recommending movement across the day to older adults with chronic pain. It is especially telling that change in overall daily activities was not associated with changes in pain intensity or pain interference. Rather, over 12 weeks, increased time spent moving at a light or greater intensity in relatively short bouts was most consistently associated with improvements in pain intensity and pain interference. Notable is the fact that time spent moving in lengthy bouts (i.e., bouts of greater than ≥20 min) was not significantly associated with improvements in pain symptoms. Taken together, these preliminary findings suggest that individuals with chronic pain may derive the most beneficial reductions in pain symptoms from a PA program focused on ambulatory movement at a light-to-moderate intensity in relatively short bouts of 5–20 min at a time. This recommendation should also be paired with efforts to accrue a sufficient volume of activity, given the immense health benefits associated with doing so (Physical Activity Guidelines Advisory Committee, 2018). Once again, we would like to emphasize the exploratory nature of these analyses, and believe our findings underscore the value of additional research explicitly testing the effects of different patterns of PA on pain symptomatology in a larger sample of older adults with chronic pain over a longer duration.

## 5 Strengths and limitations

There are several notable strengths to this study. First, we leveraged the ActivPAL physical activity monitor, which provides 24-h accelerometer data, high compliance, and reliable posture and stepping signals. (Rosenberg et al., 2020; Wu et al., 2022). Additionally, participants completed validated and widely used measures of pain intensity and pain interference with scores on these measures reflecting symptoms that were approximately one standard deviation above the national average. Half of the sample engaged in a unique PA intervention focused on moving often throughout the day, introducing greater variability in activity patterns. Finally, current studies on activity bout length and health—particularly those focused on activity fragmentation—are cross-sectional in nature (Wanigatunga et al., 2019a; Wanigatunga et al., 2019b; Schrack et al., 2019). The design of the present study allowed for a preliminary investigation of longitudinal associations between activity patterns and pain.

There are several key limitations present in these pilot trials. First, and foremost, our analyses were exploratory in nature. The sample was relatively small and comprised individuals recruited across two separate studies (though analyses accounted for study as a covariate), and results were not adjusted for multiple comparison, as at this exploratory stage we weigh the possibility of a type II error to be at least as important as a type I error (Rothman, 1990). Therefore, results should be viewed as a first step toward future longitudinal research on a larger and more diverse sample of older adults with chronic pain. Additionally, as we reported previously, (Fanning et al., 2020; Fanning et al., 2022b), there was missing accelerometer data due to battery failure resulting from overcharging of batteries; an issue that has since been addressed by ActivPAL. ActivPAL devices also do not allow for the application of intensity thresholds to score data. However, we emphasize that traditional intensity thresholds also are calibrated to stepping behavior, (Freedson et al., 1998; Copeland and Esliger, 2009). Finally, a majority of the sample was white (80.5%), female (73.2%), and college-educated (87.8%), which limits generalizability of findings to more diverse populations and reflects historical and on-going disparities in clinical trial participation. While the findings of this study are novel, they should be interpreted in the context of these limitations. Additional work is sorely needed to enhance recruitment and participation among larger and more diverse samples of older adults with chronic pain.

## 6 Conclusion

Chronic pain is very common among older adults and is both physically and psychologically debilitating. Engaging in PA is a potentially effective lifestyle medicine for managing chronic pain that is cost efficient, relatively safe with few side effects, and broadly beneficial for overall health and wellbeing beyond pain symptoms. Activity cycling often is recommended as a pacing strategy for increasing PA in the context of pain, though there is little clarity on what activity cycling means, and there is little guidance on how to implement a cycling strategy. Older adults with chronic pain may benefit by moving at light or moderate intensities in brief bouts of at least 5 min in duration. This work provides a foundation for future large scale clinical trial research investigating the impact of different PA intensities and bout durations on pain symptoms in a diverse sample of older adults with chronic pain.

## Data Availability

The original contributions presented in the study are included in the article/Supplementary Material, further inquiries can be directed to the corresponding author.
