# Supplementary material for: Associations between patterns of physical activity, pain intensity, and interference among older adults with chronic pain: a secondary analysis of two randomized controlled trials
Source: Front Aging. 2023 Jul 25;4:1216942. doi: 10.3389/fragi.2023.1216942 (PMC10411520; doi:10.3389/fragi.2023.1216942)
Supplement: Supplementary file 1 [file Table2.DOCX]

Supplementary Material

Associations between Patterns of Physical Activity, Pain Intensity, and Interference among Older Adults with Chronic Pain: A Secondary Analysis of Two Randomized Controlled Trials.

Jason Fanning, PhD,^1^ Amber K Brooks, MD, ^2^ Justin T Robison, ^1^ BS, Megan B Irby, PhD,^1^ Sherri Ford, MS,^1^ Kindia N’Dah, BS,^1^ W. Jack Rejeski, PhD^1^

*** Correspondence:**Jason Fanning
Fanninjt@wfu.edu

# Supplementary Figures and Tables

|  | **Included (N=41)** | **Insufficient Data (N=34)** | **P** |
| --- | --- | --- | --- |
| Age, yrs; M(SD) | 69.61 (6.48) | 69.32 (7.50) | .861 |
| Pain Intensity; M(SD) | 59.44 (6.59) | 60.72 (3.72) | .294 |
| Pain Interference; M(SD) | 59.82 (6.24) | 60.22 (4.75) | .761 |
| Intervention; n(%) | 23 (56.10) | 18 (52.90) | 1.000 |
| Male; n(%) | 11 (26.80) | 6 (17.60) | .414 |
| White; n(%) | 33 (80.50) | 27 (79.40) | 1.000 |
| College Educated; n(%) | 36 (87.80) | 30 (88.24) | 1.000 |
| Weight, kg; M(SD) | 98.70 (14.61) | 95.13 (15.37) | .349 |
| MORPH; n(%) | 20 (48.78) | 12 (35.5) | .254 |

Supplement Table 1: Participant information at baseline for those with and without sufficient data for inclusion. Notes: M=mean; SD=standard deviation. Independent-samples t-tests utilized for continuous variables; Fisher’s Exact tests used for count variables.

|  | **MORPH (n=20)** | **MORPH-II (n=21)** | **Overall (N=41)** |
| --- | --- | --- | --- |
| Very Light Intensity Time; M(SD) | 32.18 (17.11) | 39.58 (25.05) | 35.97 (21.60) |
| <1 minute | 28.76 (15.85) | 35.61 (20.85) | 32.27 (18.68) |
| 1-5 minutes | 2.49 (2.34) | 3.91 (4.6) | 3.22 (3.7) |
| 5-10 minutes | 0.36 (1.41) | 0.05 (0.24) | 0.2 (1) |
| 10-20 minutes | 0.58 (1.82) | 0 (0) | 0.28 (1.29) |
| 20+ minutes^a^ | - | - | - |
| Light Intensity Time; M(SD) | 25.32 (15.04) | 25.41 (14.1) | 25.37 (14.38) |
| <1 minute | 17.55 (8.87) | 20.3 (10.72) | 18.96 (9.84) |
| 1-5 minutes | 6.75 (6.44) | 4.45 (3.33) | 5.57 (5.16) |
| 5-10 minutes | 0.8 (1.58) | 0.39 (1.17) | 0.59 (1.38) |
| 10-20 minutes | 0 (0) | 0.27 (0.86) | 0.14 (0.62) |
| 20+ minutes | 0.22 (0.97) | 0 (0) | 0.11 (0.68) |
| Moderate Intensity Time; M(SD) | 6.92 (7.76) | 5.86 (6.57) | 6.38 (7.10) |
| <1 minute | 3.27 (2.41) | 3.81 (4.89) | 3.54 (3.85) |
| 1-5 minutes | 1.95 (3.58) | 1.18 (1.47) | 1.55 (2.7) |
| 5-10 minutes | 0.3 (0.78) | 0.08 (0.38) | 0.19 (0.61) |
| 10-20 minutes | 0.43 (0.91) | 0.47 (1.68) | 0.45 (1.34) |
| 20+ minutes | 0.98 (4.39) | 0.31 (1) | 0.64 (3.12) |

Supplement Table 2: Average time spent in activity bouts of varying duration at baseline. Notes: M=mean; SD=standard deviation; s/m=steps per minute; very light intensity time = average daily minutes spent stepping at <75 s/m; light intensity time = average daily minutes spent stepping at 75-100 s/m; moderate intensity time = average daily minutes spent stepping at 100-125 s/m.
